# Multiple Electronic Phases Coexisting under Inhomogeneous Strains in the Correlated Insulator

**DOI:** 10.1002/advs.202300789

**Published:** 2023-04-25

**Authors:** Baofei Hou, Yu Zhang, Teng Zhang, Jizheng Wu, Quanzhen Zhang, Xu Han, Zeping Huang, Yaoyao Chen, Hongyan Ji, Tingting Wang, Liwei Liu, Chen Si, Hong‐Jun Gao, Yeliang Wang

**Affiliations:** ^1^ School of Integrated Circuits and Electronics MIIT Key Laboratory for Low‐Dimensional Quantum Structure and Devices Beijing Institute of Technology Beijing 100081 China; ^2^ Advanced Research Institute of Multidisciplinary Sciences Beijing Institute of Technology Beijing 100081 China; ^3^ School of Materials Science and Engineering Beihang University Beijing 100191 China; ^4^ Center for Integrated Computational Materials Engineering International Research Institute for Multidisciplinary Science Beihang University Beijing 100191 China; ^5^ Institute of Physics Chinese Academy of Sciences Beijing 100190 China

**Keywords:** charge‐density‐wave, correlated insulator, inhomogeneous strain, scanning tunneling microscopy

## Abstract

Monolayer transition metal dichalcogenides (TMDs) can host exotic phenomena such as correlated insulating and charge‐density‐wave (CDW) phases. Such properties are strongly dependent on the precise atomic arrangements. Strain, as an effective tuning parameter in atomic arrangements, has been widely used for tailoring material's structures and related properties, yet to date, a convincing demonstration of strain‐induced dedicate phase transition at nanometer scale in monolayer TMDs has been lacking. Here, a strain engineering technique is developed to controllably introduce out‐of‐plane atomic deformations in monolayer CDW material 1T‐NbSe_2_. The scanning tunneling microscopy and spectroscopy (STM and STS) measurements, accompanied by first‐principles calculations, demonstrate that the CDW phase of 1T‐NbSe_2_ can survive under both tensile and compressive strains even up to 5%. Moreover, significant strain‐induced phase transitions are observed, i.e., tensile (compressive) strains can drive 1T‐NbSe_2_ from an intrinsic‐correlated insulator into a band insulator (metal). Furthermore, experimental evidence of the multiple electronic phase coexistence at the nanoscale is provided. The results shed new lights on the strain engineering of correlated insulator and useful for design and development of strain‐related nanodevices.

## Graphical abstract

In this work, a strain engineering technique is developed to controllably introduce an inhomogeneous strain in monolayer 1T‐NbSe_2_, a correlated insulator with charge density wave, and verify the existence of the multiple electronic‐phase coexisting signature at the nanoscale with the combination of scanning tunneling microscopy/spectroscopy measurements and first‐principles calculations.

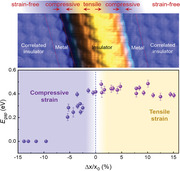

## 1 Introduction

Tailoring the electronic structures of a material by subjecting it to strain has been widely utilized in both scientific research and applications. Especially for the atomically thin materials that are capable of withstanding extremely large mechanical deformation before rupture, strain engineering is intensely pursued to manipulate the electronic structures over a wide range, thus offering a unique opportunity to achieve high‐performance devices.^[^ ^1^, ^2^, ^3^, ^4^, ^5^ ^]^ For example, strains are expected to introduce giant pseudomagnetic fields of over 300 T and flat bands in graphene,^[^ ^6^, ^7^, ^8^, ^9^, ^10^ ^]^ as well as lead to significant topological or magnetic phase transitions in low‐dimensional systems.^[^ ^11^, ^12^, ^13^ ^]^ Although central to our understanding of strain engineering, a direct characterization of strain‐driven local phase transition in correlated electronic systems has been experimentally elusive yet to date, especially for the coexistence of multiple electronic phases induced by inhomogeneous strains at the nanoscale. In addition, the technique for controllable generation of local strains in low‐dimensional systems is still lacking, which needs further exploration.

Monolayer group‐V transition metal dichalcogenides (TMDs) in 1T phase, such as 1T‐NbSe_2_, 1T‐TaS_2_, and 1T‐TaSe_2_, have evoked great interest in recent years, owing to the existence of rich quantum phases.^[^ ^14^, ^15^, ^16^, ^17^, ^18^, ^19^ ^]^ Specifically, atomic lattices of monolayer 1T‐TMDs usually experience a charge‐density‐wave (CDW) phase transition upon cooling, which spontaneously generates an extremely narrow flat band at the Fermi level. Strong on‐site Coulomb repulsion *U* further drives the flat band splitting into upper and lower Hubbard bands (UHB and LHB), thus resulting in a correlated insulating phase.^[^ ^14^, ^15^, ^16^, ^17^ ^]^ Moreover, the energy and the splitting behavior of such a flat band is predicted to be extremely susceptible to the perturbation or tiny distortions of atomic arrangements.^[^ ^20^, ^21^, ^22^, ^23^, ^24^, ^25^ ^]^ Therefore, monolayer 1T‐TMDs provide ideal platforms to investigate the strain‐induced rich‐correlated phase transitions.

In this work, we develop an in‐situ strain engineering technique to controllably introduce locally strained structures in monolayer 1T‐NbSe_2_, and report direct experimental evidence of the strain‐induced correlated phase transitions. Our scanning tunneling microscopy (STM) and spectroscopy (STS) measurements, accompanied by first‐principles calculations, demonstrate that the CDW phase of monolayer 1T‐NbSe_2_ can survive under both tensile and compressive strains even up to 5%. We also observe significant strain‐induced phase transitions, i.e., a tensile strain drives 1T‐NbSe_2_ from an intrinsic correlated insulator into a band insulator, while a compressive strain results in an insulator‐metal transition. Unexpectedly, all these phases can coexist at the nanoscale. Our results provide convincing demonstration of the strain engineering in correlated physics regimes.

## 2 Results and Discussion

**Figure 1:**
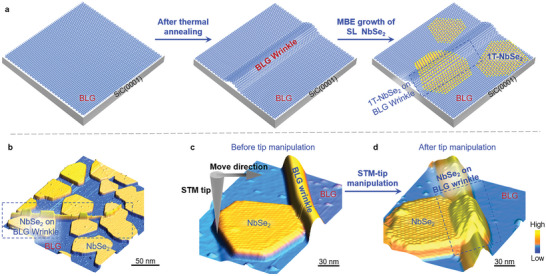
Nanoscale control of strains in monolayer 1T‐NbSe_2_. a) Schematic of the MBE growth of monolayer 1T‐NbSe_2_ islands on SiC(0001) substrates that are covered with BLG wrinkles. The BLG wrinkles are controllably generated when the annealing temperature exceeds 1350 °C. The NbSe_2_ islands are then epitaxially grown on and off the BLG wrinkles by directly evaporating Nb and Se atoms under a rich Se environment. b) Representative STM image of monolayer 1T‐NbSe_2_ islands on and off the BLG wrinkles (*V*_s_ = –2.0 V, *I_t_* = 5 pA). The BLG wrinkles can be clearly identified as one‐dimensional protrusions with the apparent heights, exhibiting in the STM images of about 1 nm. c,d) Nanoscale control of monolayer 1T‐NbSe_2_ islands on and off the BLG wrinkles via an in‐situ STM manipulation technique (*V*_s_ = –1.0 V, *I_t_* = 5 pA).

To realize strained monolayer 1T‐NbSe_2_ structures, in our experiments, we first introduce wrinkles of bilayer graphene (BLG) on SiC(0001) via an annealing process under the temperature exceeding 1350 °C and a fast cooling process, owing to the differing thermal expansion coefficients between graphene and SiC.^[^ ^26^, ^27^ ^]^ And then, monolayer 1T‐NbSe_2_ islands were synthesized onto BLG/SiC substrates via a molecular beam epitaxy (MBE) method,^[^ ^28^, ^29^ ^]^ as schematically depicted in **Figure** **1**a. From the large‐scale STM image of an as‐grown sample shown in Figure 1b, we can find that monolayer 1T‐NbSe_2_ islands are expected to be triggered arbitrarily on BLG, regardless of the wrinkles, thus yielding a certain amount of monolayer 1T‐NbSe_2_ islands overlying onto the BLG wrinkles and following their bending structures. Therefore, monolayer 1T‐NbSe_2_ islands with one‐dimensional strains are spontaneously achieved (see Figure S1, Supporting Information).

For the monolayer 1T‐NbSe_2_ islands off the BLG wrinkles, such strained structures can also be artificially generated via an explored in‐situ manipulating technique at the nanoscale. Specifically, we first bring the STM tip approaching to BLG substrate by increasing the tunneling current over 1 nA and decreasing the bias voltage to 0.05 V, and then, set the trajectory of the STM tip perpendicular to the edge of a monolayer 1T‐NbSe_2_ island.^[^ ^28^ ^]^ During this process, the island can be controllably pushed to slide on BLG and finally cross the wrinkles, as demonstrated in Figure 1c,d of the representative STM images before and after the STM‐tip manipulation. By this means, the strained monolayer 1T‐NbSe_2_ structures are realized in a controlled manner.

**Figure 2:**
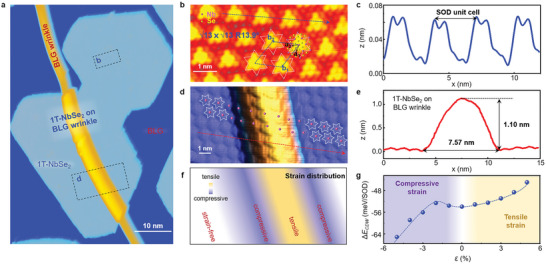
Atomic structures of monolayer 1T‐NbSe_2_ under strains. a) Large‐scale STM image of monolayer 1T‐NbSe_2_ islands on BLG wrinkles (*V*_s_ = –2.0 V, *I_t_* = 5 pA). b) Zoomed‐in atomic‐resolution STM image of monolayer NbSe_2_ on pristine BLG (*V*_s_ = –1.0 V, *I_t_* = 2 nA). a_1_/a_2_ and b_1_/b_2_ represent the basis vectors of 1 × 1 atomic lattices and √13 × √13 CDW lattices, respectively. c) The height profile along the blue arrow in panel b. d) Zoomed‐in atomic‐resolution STM image of monolayer NbSe_2_ on BLG wrinkle (*V*_s_ = –1.0 V, *I_t_* = 1.5 nA). The center Nb atom in each SOD is marked by the pink dot. e) The height profile along the red arrow in panel d, demonstrating the apparent height and width of the wrinkle in the STM image are about 1.10 and 7.57 nm, respectively. f) Schematic of the strain distribution. g) CDW formation energy Δ*E*_CDW_ as a function of the strain *ε*. Positive and negative *ε* represent tensile and compressive strains, respectively. The dashed line is a guide to the eyes.

Now, we concentrate on the precise atomic structures of monolayer 1T‐NbSe_2_ on and off BLG wrinkles. **Figure** **2**a shows a representative STM image of a monolayer 1T‐NbSe_2_ island on a BLG wrinkle acquired at the liquid helium temperature. A close examination of the atomic structures offs the wrinkle shown in Figure 2b, as well as the corresponding height profile in Figure 2c reveals that the top Se atoms dominate the topography of 1T‐NbSe_2_ in CDW phase, exhibiting a √13 × √13 triangular superlattice.^[^ ^30^ ^]^ The basic element of CDW phase is the so‐called star‐of‐David (SOD) cluster that is composed of 13 Nb atoms and 26 Se atoms, where 12 surrounding Nb atoms contracting toward one central Nb atom, as depicted in Figure 2b.

The atomic‐resolution STM image of monolayer 1T‐NbSe_2_ on a BLG wrinkle and the corresponding line profile are shown in Figure 2d,e, respectively. Such large curvature and prominent out‐of‐plane configuration of 1T‐NbSe_2_ wrinkles make them ideal candidates for the realization of local strains. From Figure 2d, we can find that the three‐dimensional distance along the wrinkle between the nearest‐neighbor SOD clusters, defined as *x*, are spatially inhomogeneous, implying the existence of complex strain fields in the vicinity of 1T‐NbSe_2_ wrinkles. Here, we should emphasize that both the electronic states and topographic features contribute to the measured apparent height in STM images, and hence, we can only obtain the relative profile heights of the 1T‐NbSe_2_ wrinkles as well as roughly estimate the strain strength under the same experimental condition for comparison. It is worth noting that the well‐ordered SOD clusters of 1T‐NbSe_2_ can always be observed around all the studied wrinkles as the temperature varying from 4.2 to 100 K, regardless of their shapes or orientations (see Figure S2, Supporting Information). These results undoubtedly demonstrate that the CDW phase of monolayer 1T‐NbSe_2_ is quite robust under external strains.

To provide a comprehensive understanding of our experiments, we first roughly captured the strain distribution in the vicinity of monolayer 1T‐NbSe_2_ wrinkles in Figure 2f, based on values Δ*x*/*x*_0_ on the SOD clusters from Figure 2d. Here, we define Δ*x* as the difference between *x* and *x*_0_, and *x*_0_ is the intrinsic superlattice constant of monolayer 1T‐NbSe_2_ in the CDW phase without strain (more details are given in Figure S3, Supporting Information). As we can see, the atomic lattice prefers to be tensile at the center of the wrinkles, while compressive at the two sides, which is maybe because the BLG substrate that helps monolayer 1T‐NbSe_2_ maintain such a strain field. Our density functional theory (DFT) calculations further demonstrate that the CDW phase of monolayer 1T‐NbSe_2_ can survive under both tensile and compressive strains even up to 5%, as shown in Figure 2g.

**Figure 3:**
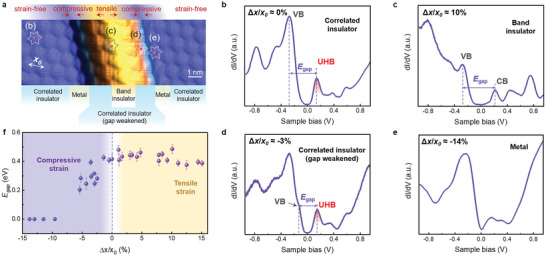
Electronic structures of monolayer 1T‐NbSe_2_ under strains. a) Typical STM image of monolayer NbSe_2_ islands on BLG wrinkles (*V*_s_ = –1.0 V, *I_t_* = 1.5 nA). The intrinsic superlattice constant of monolayer NbSe_2_ in the CDW phase without strain *x*_0_ is marked by the double‐headed arrow. The multiphase coexisting behavior induced by inhomogeneous strains is marked in the panel. b–e) Typical STS spectra acquired at the center of SOD clusters marked in panel a (*V*_s_ = –1.0 V, *I_t_* = 1.5 nA). Δ*x* is the difference between *x* and *x*_0_, where *x* is the measured average superlattice constant in three dimensions by considering the nearest two neighbors of CDW clusters. Δ*x* > 0 and Δ*x* < 0 represent tensile and compressive strains, respectively. The energy gap *E*_gap_ can be acquired from the STS spectra, as marked in the panels. f) The average energy gap *E*_gap_ and the corresponding error bars as a function of Δ*x*/*x*_0_.

The electronic structures of monolayer 1T‐NbSe_2_ can be strongly tuned by local strains. Here, we carried out the STS measurements in the vicinity of monolayer 1T‐NbSe_2_ wrinkles (**Figure** **3**a) under open feedback conditions by a standard lock‐in amplifier with a modulation frequency of 973 Hz and a modulation amplitude of 5 mV at 4.2 K. Figure 3b–e shows typical STS spectra recorded at the center of SOD clusters on and off a monolayer 1T‐NbSe_2_ wrinkle marked in Figure 3a, which can directly reflect the local density of states (LDOS). Away from the wrinkle (Figure 3b), the STS spectra show a low‐intensity signature within an energy range, implying that pristine monolayer 1T‐NbSe_2_ is a correlated insulator when taking the tunneling mechanism of STS measurements into consideration.^[^ ^31^, ^32^ ^]^ Moreover, there are several pronounced LDOS peaks exhibited in STS spectra, with their intensities varying as the periodicity of CDW order. Specifically, the peak at 0.16 eV originates from the spin‐polarized UHB (Figure S4, Supporting Information), the peak at –0.28 eV is attributed to the high mixture of the LHB weight and the valance band (VB), and the peak at –0.78 eV mainly derives from the LHB weight. These features are well consistent with previous experimental studies^[^ ^33^, ^34^, ^35^ ^]^ and our DFT calculations. In addition, we can easily rule out the influence of BLG substrate on the electronic properties of monolayer 1T‐NbSe_2_, owing to the weak electronic coupling between 1T‐NbSe_2_ and graphene (Figures S4–S6, Supporting Information).

The STS spectra acquired in the vicinity of monolayer 1T‐NbSe_2_ wrinkles exhibit significant site‐dependent features. At the center of a wrinkle, i.e., monolayer 1T‐NbSe_2_ undergoes a tensile strain, the UHB vanishes, instead a DOS peak appears at about 0.20 eV, as marked in Figure 3c. In contrast, at the sides of a wrinkle, i.e., monolayer 1T‐NbSe_2_ undergoes a compressive strain, the energy of the VB shifts upward, and further results in either a suppression of the energy gap shown in Figure 3d, or a metallic state shown in Figure 3e, depending on the strength of the compressive strain at the measured sites (high‐resolution STS spectra are given in Figure S7, Supporting Information). Such features are quite distinct from the STS spectra recorded on bare BLG wrinkles shown in Figure S6 (Supporting Information), demonstrating that the topmost 1T‐NbSe_2_ layer dominates the observed phase transitions.^[^ ^36^, ^37^, ^38^ ^]^

Moreover, we summarize the evolution of the energy gap *E*_gap_ as a function of Δ*x*/*x*_0_ in Figure 3f, extracting from the spatially resolved STS spectra recorded across different monolayer 1T‐NbSe_2_ wrinkles by using different STM tips. In our experiments, *E*_gap_ is defined as the energy separation between the two DOS peaks close to and separated on either side of the Fermi level when the spectrum exhibits an insulating state, and *E*_gap_ is set as zero when the spectrum shows metallic behavior. From Figure 3f, we can see that there is an obvious increase of *E*_gap_ by applying a tensile strain, while the *E*_gap_ decreases and finally vanishes by applying a compressive strain.

**Figure 4:**
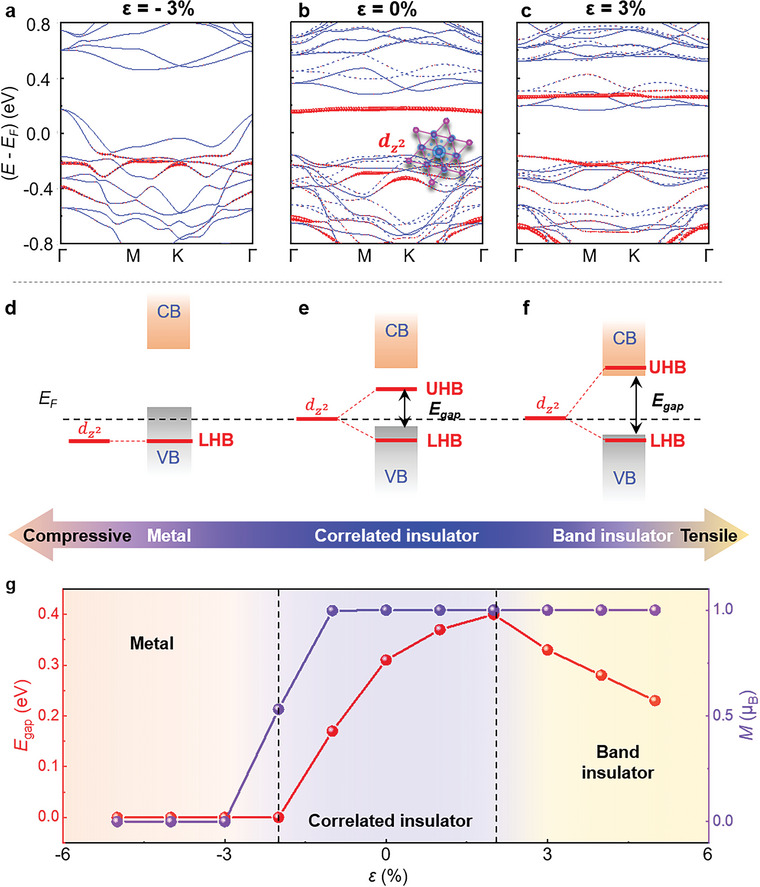
Strain‐tuned band structures of monolayer 1T‐NbSe_2_ in the CDW phase. a–c) DFT calculations of the band structures of 1T‐NbSe_2_ in the CDW phase under the strains *ε* = –3%, 0%, and 3%, respectively. The dashed and solid blue lines represent the up and down spin bands, respectively. The $d_{z^{2}}$ orbital is highlighted by the red dots, and the size of the dots is proportional to the Nb central‐atom $d_{z^{2}-r^{2}}$ orbital character within each SOD cluster. The real‐space charge density distribution of the $d_{z^{2}}$ orbital is given in the inset of panel b. d–f) Corresponding schematic energy level diagrams of 1T‐NbSe_2_ in the CDW phase under the strains *ε* = –3%, 0%, and 3%, respectively. Without consideration of the strong electronic correlation, there is a flat band $d_{z^{2}}$, which is mainly contributed by the central Nb atom of each SOD cluster. Once the flat band is at the Fermi level, strong electronic correlation further splits the flat band into UHB and LHB. g) Theoretical calculation of the energy gap and the total magnetic moment per SOD cluster of 1T‐NbSe_2_ in the CDW phase as a function of *ε*.

To understand the above experimental phenomena, we carried out DFT calculations of the strain‐induced band structures in monolayer 1T‐NbSe_2_ at the CDW phase. **Figure** **4**a–c shows the band structures of monolayer 1T‐NbSe_2_ under strains *ε* = –3%, 0%, and 3%, respectively, complemented by the corresponding schematic energy level diagrams depicted in Figure 4d–f (more results are given in Figure S8, Supporting Information). As we can see, the band structures of monolayer 1T‐NbSe_2_, especially the $d_{z^{2}}$ orbital highlighted by the red dots, can be strongly modified under strains. For example, by applying a tensile strain, the enhanced on‐site Coulomb repulsion of the $d_{z^{2}}$‐derived flat band drives the isolated UHB component shifts upward in energy, and finally merges into the conduction band (CB) when the tensile strain reaches 3%, yielding a phase transition from the correlated insulator to the band insulator (Figure 4c). In contrast, a compressive strain applying to monolayer 1T‐NbSe_2_ can efficiently drive the VB shifting upward, as well as suppress the Coulomb repulsion of the $d_{z^{2}}$‐derived flat band. As the compressive strain exceeding –2%, the $d_{z^{2}}$ orbital highly hybridizes with the VB, resulting in the phase transition from the correlated insulator to the metallic phase (Figure 4a).

Figure 4g summarizes the calculated energy gap, marked in Figure 4d–f, as a function of the strain *ε*. In the correlated insulating regime under a slight strain within ±2%, a larger lattice constant of monolayer 1T‐NbSe_2_ leads to an enlarged correlated gap. Moreover, a large tensile strain induces a phase transition from the correlated insulator into the band insulator, while a large compressive strain induces a phase transition from the correlated insulator into the metal. These results are well consistent with our experimental phenomena shown in Figure 3. Therefore, our experiments not only provide the direct experimental evidence of the strain‐induced correlated phase transitions but also verify the ability of multiphase coexistence at the nanoscale, including correlated insulator, band insulator, and metal. With the combination of STS spectra and DFT results, we can roughly estimate that the maximal local strain in our studied monolayer 1T‐NbSe_2_ wrinkles is within ±3%. In addition, the total magnetic moment per SOD cluster is also calculated in Figure 4g, demonstrating the existence of nonzero magnetic moments in insulating monolayer 1T‐NbSe_2_ with the CDW phase.

## 3 Conclusion

In summary, we systemically study the strain‐induced phase transition in correlated insulator monolayer materials 1T‐NbSe_2_ at nanometer scale. By developing a strain engineering technique to controllably introduce local strains in monolayer 1T‐NbSe_2_, we find that the CDW phase can survive under both tensile and compressive strains. Moreover, a tensile strain can drive monolayer 1T‐NbSe_2_ from an intrinsic correlated insulator into a band insulator, while a compressive strain results in an obvious metal phase, and all these phases can coexist at the nanoscale. Our results shed new lights on the strain engineer of correlated phase transition materials and are useful for design and development of strain‐related nanodevices.

## 4 Experimental Section

### Sample Preparation

The sample preparation and STM measurements were carried out by a custom‐designed Unisoku STM system (USM‐1300). First, bilayer graphene (BLG) was obtained by thermal decomposition of 4H‐SiC(0001) at 1200 °C for 45 min. And then, BLG‐covered 4H‐SiC(0001) was thermal annealed at the temperature higher than 1300 °C for 20 min to generate BLG wrinkles. Next, the NbSe_2_ islands were epitaxially grown on graphene/SiC(0001) substrate by evaporating Nb and Se from an electron beam evaporator and a Knudsen cell evaporator, respectively. The flux ratio of Nb and Se was ≈1:20, in order to guarantee a rich Se environment. The growth rate of NbSe_2_ was 0.002 ML min^−1^. The graphene/SiC(0001) substrate was maintained at 500 °C during the growth, followed by a postannealing process at 400 °C for 20 min.

### STM Measurements

The STM and STS measurements were performed in the ultrahigh vacuum chamber (≈10^−11^ Torr) with constant‐current scanning mode. The experiments were acquired at the temperature of 4.2 K. An electrochemically etched tungsten tip was used as the STM probe, which was calibrated by using a standard graphene lattice, a Si (111)‐(7×7) lattice, and a Ag (111) surface. The STS measurements were taken by a standard lock‐in technique with the bias modulation of 5 mV at 973 Hz.

### First‐Principles Calculations

The calculations were performed in the framework of the density functional theory (DFT) using projector‐augmented wave (PAW) potentials, as implemented in the Vienna ab initio simulation package (VASP). To describe the electron correlations of Nb‐*d* orbitals, the GGA+*U* approach with *U* = 2 eV was used, which could nicely reproduce the experimental‐correlated gap of monolayer 1T‐NbSe_2_. The energy cutoff for the plane‐wave basis expansion was set to 500 eV. To simulate the monolayer model of 1T‐NbSe_2_, a vacuum layer of 20 Å is used. The Brillouin zone is integrated with a Γ‐centered *k* meshes with sufficient *k*‐point densities. All the structures are relaxed until the remaining force acting on each atom is less than 0.01 eV Å^−1^. Note that although a fixed *U* value is used in the calculations of 1T‐NbSe_2_ at all strain levels, the effective *U* acting on the d orbital of the central Nb atom, *U*_eff_, changes with strain. This is because the degree of *p*–*d* hybridization between the central Nb atom and its surrounding Se atoms will be modified by strain.

## Conflict of Interest

The authors declare no conflict of interest.

## Supporting information

Supporting Information

## Data Availability

The data that support the findings of this study are available from the corresponding author upon reasonable request.
