# Supplementary material for: Multiple Electronic Phases Coexisting under Inhomogeneous Strains in the Correlated Insulator
Source: Adv Sci (Weinh). 2023 Apr 25;10(19):2300789. doi: 10.1002/advs.202300789 (PMC10323623; doi:10.1002/advs.202300789)
Supplement: Supplementary file 1 — Supporting Information [file ADVS-10-2300789-s001.pdf]

ADVANCED SCIENCE
Open Access

# Supporting Information

for *Adv. Sci.*, DOI 10.1002/advs.202300789

Multiple Electronic Phases Coexisting under Inhomogeneous Strains in the Correlated Insulator

*Baofei Hou, Yu Zhang*, Teng Zhang, Jizheng Wu, Quanzhen Zhang, Xu Han, Zeping Huang, Yaoyao Chen, Hongyan Ji, Tingting Wang, Liwei Liu, Chen Si*, Hong-Jun Gao and Yeliang Wang**

# Supporting Information

## Multiple electronic phases coexisting under inhomogeneous strains in the correlated insulator

*Baofei Hou, Yu Zhang*, Teng Zhang, Jizheng Wu, Quanzhen Zhang, Xu Han, Zeping Huang, Yaoyao Chen, Hongyan Ji, Tingting Wang, Liwei Liu, Chen Si*, Hong-Jun Gao, and Yeliang Wang**

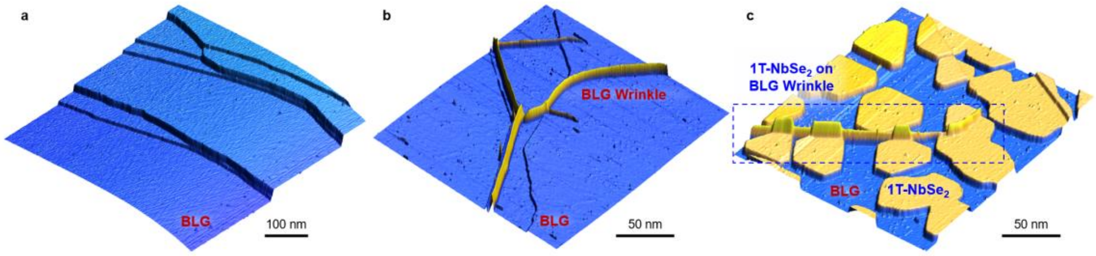


**Figure S1**. a) Large-scale STM image of well-defined BLG on SiC(0001) substrate ($V_s$ = -1.5 V, $I_t$ = 100 pA). b) Large-scale STM image of BLG wrinkles after thermal annealing process ($V_s$ = -1.5 V, $I_t$ = 30 pA). c) Representative STM image of monolayer 1T-$NbSe_2$ islands on and off the BLG wrinkles ($V_s$ = -2.0 V, $I_t$ = 5 pA).

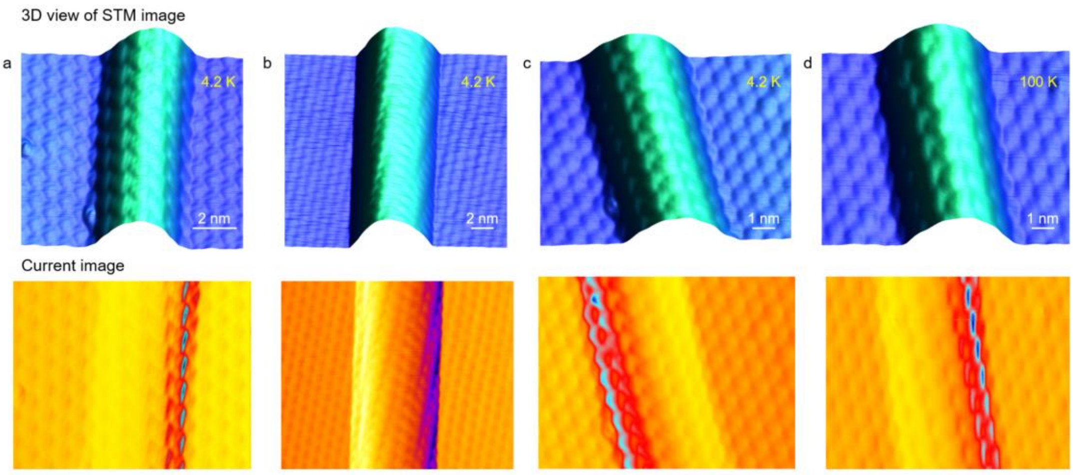


**Figure S2**. STM images and the corresponding current images of monolayer 1T-$NbSe_2$ islands on BLG wrinkles measured under 4.2 K and 100 K. The SOD clusters are always visible on and off all the wrinkles, independent of the orientation of the wrinkle.

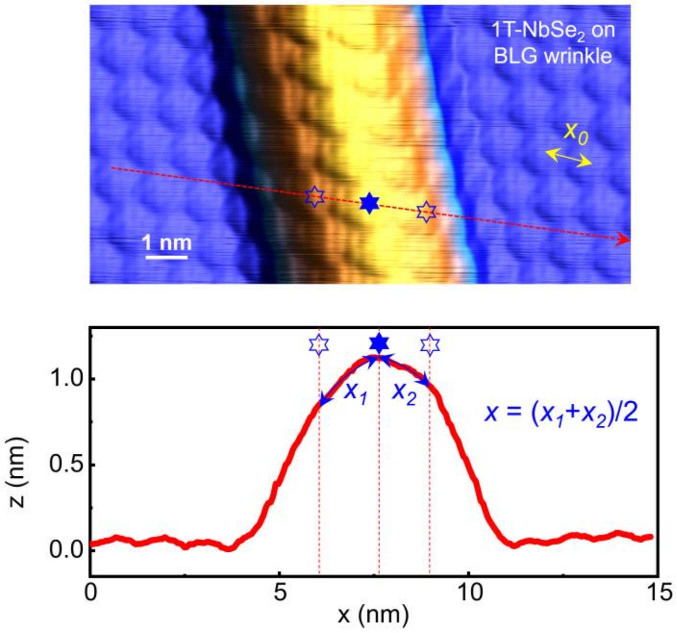


**Figure S3**. Method of determining $\Delta x/x$. $\Delta x$ is the difference between $x$ and $x_0$, where $x_0$ is defined as the intrinsic CDW superlattice constant of monolayer 1T-$NbSe_2$, and $x$ is the average arc length of the nearest two neighbors of SOD clusters acquired from the arc length in a height profile. The red dashed lines roughly donate the sites of SOD centers in monolayer 1T-$NbSe_2$.

Figure S4a exhibits a typical STM image of a 1T/1H-$NbSe_2$ vertical heterostructure on bilayer graphene. The topmost monolayer 1T-$NbSe_2$ shows a $\sqrt{13} \times \sqrt{13}\ R30^{\circ}$ triangular superlattice, while the underlying monolayer 1H-$NbSe_2$ shows a $3 \times 3$ triangular superlattice aligned with the atomic lattice, as depicted in Figure S4b and S4c, respectively. From Figure S4d of the STS spectra acquired on the 1T/1H-$NbSe_2$ vertical heterostructure, we can observe an obvious zero-bias density-of-states peak, i.e., the Kondo resonance peak, which is generated by a local spin in each SOD of monolayer 1T-$NbSe_2$ coupling to itinerant electrons in a metal monolayer 1H-$NbSe_2$. Therefore, our experiments provide evidences that there is a local magnetic moment in each SOD of monolayer 1T-$NbSe_2$, and simultaneously, 1T-$NbSe_2$/1H-$NbSe_2$ vertical heterostructure can be regarded as a Kondo lattice.

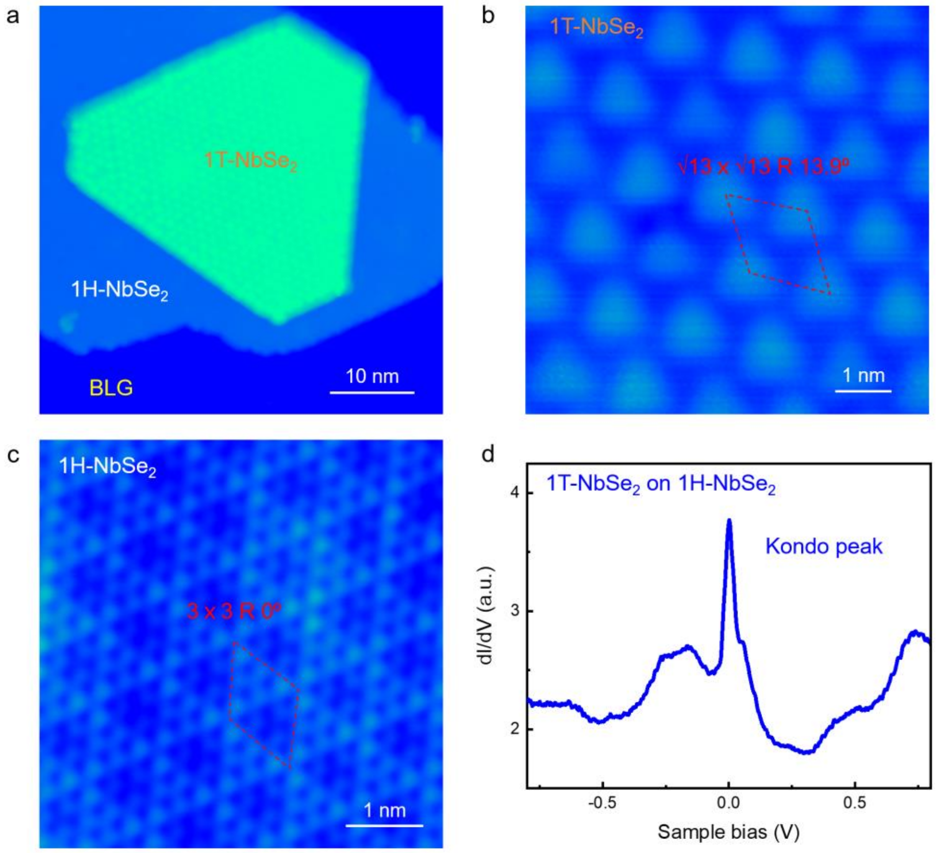


**Figure S4**. Electronic properties of 1T/1H-$NbSe_2$ vertical heterostructures. a) Typical STM image of a 1T/1H-$NbSe_2$ vertical heterostructure on bilayer graphene. b) STM image of the topmost monolayer 1T-$NbSe_2$, exhibiting a $\sqrt{13} \times \sqrt{13}\ R30^{\circ}$ triangular superlattice. c) STM image of the underlying monolayer 1H-$NbSe_2$, exhibiting a $3 \times 3$ triangular superlattice aligned with the atomic lattice. d) STS spectra acquired on the 1T/1H-$NbSe_2$ vertical heterostructure. There is an obvious Kondo resonance peak, which is generated by a local spin in each SOD of monolayer 1T-$NbSe_2$ coupling to itinerant electrons in a metal monolayer 1H-$NbSe_2$, demonstrating the existence of a magnetic moment in each SOD of monolayer 1T-$NbSe_2$ and the construction of a Kondo lattice in 1T-$NbSe_2$/1H-$NbSe_2$ vertical heterostructures.

The BLG-covered SiC(0001) substrate has almost no influence on the electronic properties of monolayer 1T-$NbSe_2$ for the following three reasons. Firstly, the STS spectra recorded on pristine monolayer 1T-$NbSe_2$ show a correlated insulating state, and all the peaks are consistent with the theoretical calculations of pristine monolayer 1T-$NbSe_2$. Secondly, although there are itinerant electrons in BLG, no Kondo resonance peak appears for monolayer 1T-$NbSe_2$ on BLG substrate (each SOD of monolayer 1T-$NbSe_2$ hosts a local magnetic moment). In contrast, the 1T/1H-$NbSe_2$ heterostructures exhibit obvious Kondo resonance peak (Figure S4). From the height profile shown in Figure S5, we can find out that the interlayer spacing between 1T-$NbSe_2$ and graphene is about 0.74 nm, much larger than that between 1T-$NbSe_2$ and 1H-$NbSe_2$ of about 0.54 nm. Therefore, the electronic coupling between monolayer 1T-$NbSe_2$ and BLG substrate is extremely weak. Thirdly, the STS spectra recorded on the boundary of monolayer 1T-$NbSe_2$ wrinkles show a metallic state, completely inconsistent with the STS spectra of BLG wrinkles (Figures S6 and S7). Therefore, we can rule out the influence of BLG-covered SiC(0001) substrate on the electronic properties of monolayer 1T-$NbSe_2$ in our experiments.

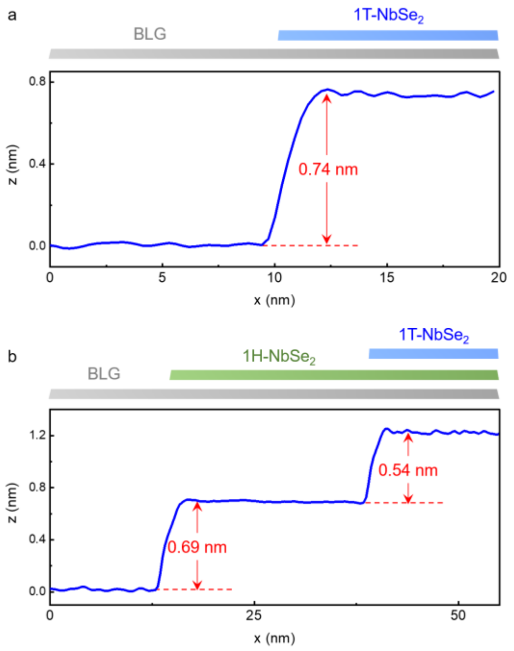


**Figure S5**. Height profiles along the 1T-$NbSe_2$/BLG and 1T-$NbSe_2$/1H-$NbSe_2$/BLG from STM images, respectively.

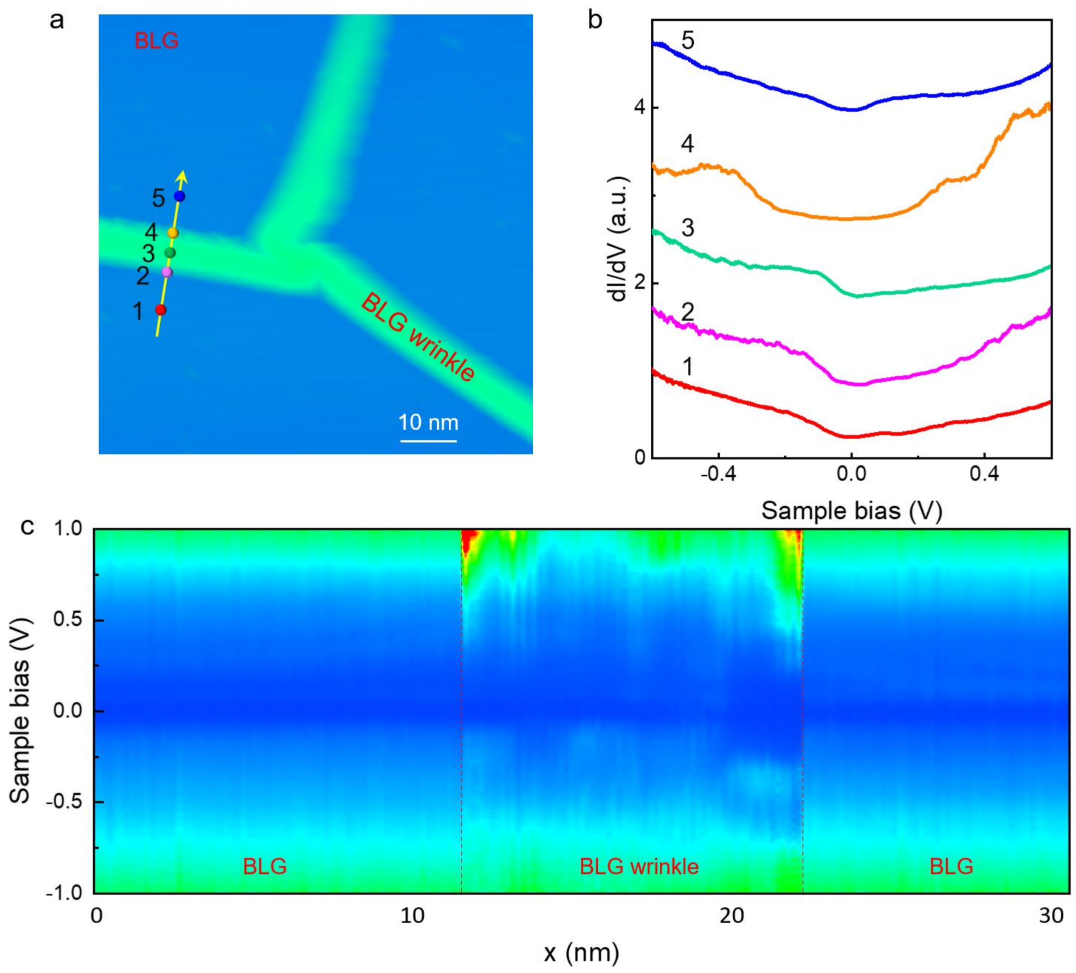


**Figure S6**. Electronic properties of BLG wrinkles. a) Representative STM image of a BLG wrinkle. b) Typical STS spectra recorded on and off the BLG wrinkle, as marked in panel a. c) Spatially resolved STS spectra recorded across the BLG wrinkle along the yellow arrow in panel a.

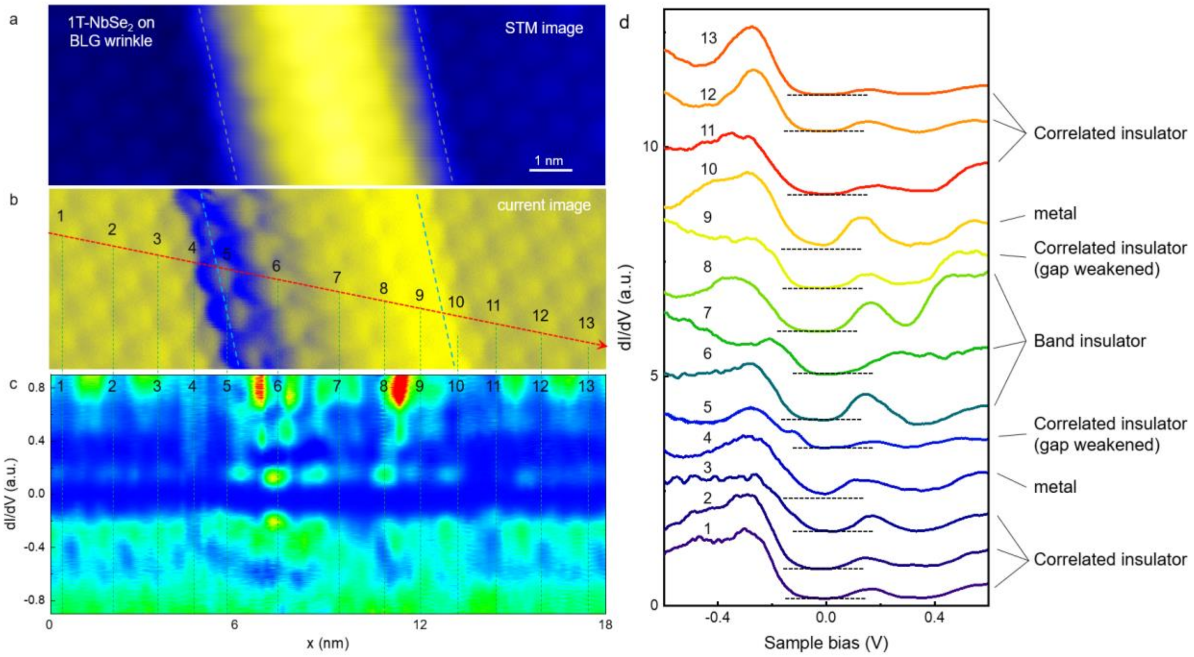


**Figure S7**. STS spectra across the monolayer 1T-$NbSe_2$ wrinkle. a) STM image of a monolayer 1T-$NbSe_2$ wrinkle. b) Corresponding current image. c) STS spectra recorded across the monolayer 1T-$NbSe_2$ wrinkle along the red arrow in panel b. d) Typical STS spectra at the sites marked in panel b.

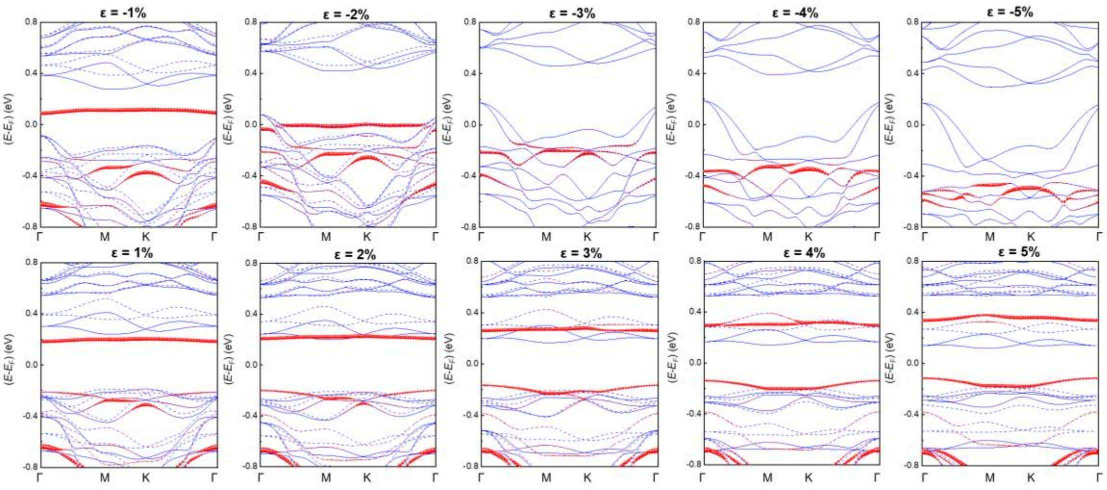


**Figure S8**. DFT calculations of the band structures of monolayer 1T-$NbSe_2$ in the CDW phase under various tensile and compressive strains.
